# Multichannel Electrical Impedance Spectroscopy Analyzer with Microfluidic Sensors †

**DOI:** 10.3390/s19081891

**Published:** 2019-04-20

**Authors:** Jaan Ojarand, Mart Min, Ants Koel

**Affiliations:** Thomas Johann Seebeck Department of Electronics, Tallinn University of Technology, 19086 Tallinn, Estonia; mart.min@taltech.ee (M.M.); ants.koel@taltech.ee (A.K.)

**Keywords:** impedance spectroscopy, microfluidic sensor, non-faradaic, front-end electronics, differential measurement, calibration, label-free detection, lab-on-a-chip

## Abstract

Impedance spectroscopy is a common approach in assessing passive electrical properties of biological matter. However, several problems appear in microfluidic devices in connection with the requirement for high sensitivity of signal acquisition from small volume sensors. The developed compact and inexpensive analyzer provides impedance spectroscopy measurement from three sensors, both connected in direct and differential modes. Measurement deficiencies are reduced with a novel design of sensors, measurement method, optimized electronics, signal processing, and mechanical design of the analyzer. Proposed solutions are targeted to the creation of reliable point-of-care (POC) diagnostic and monitoring appliances, including lab-on-a-chip type devices in the next steps of development. The test results show the good working ability of the developed analyzer; however, also limitations and problems that require attention and further improvement are appointed.

## 1. Introduction

The current article is an extended revision of the recent conference paper [1], in which impedimetric microfluidic devices target facilitation of dielectric and conductivity measurements of small fluidic volumes. The measuring fluid may be an electrolyte cell suspension or microparticles, a biochemical solution of metabolites, proteins, and nucleic acids [2,3]. Most biosensors require a labeling substance attached to the target; during readout, the amount of labeling substance is detected, and it is assumed to correspond to the number of bound targets [4]. Labels can be, for example, fluorophores detectable with optical methods. Label-based methods are sensitive and widely used, but label-free sensors have several advantages, including simplicity and speed of the measurement procedure that enables real-time monitoring of the binding reaction, thus giving access to the kinetic and thermodynamic parameters [5]. Although optical label-free detection is introduced [5], electrical label-free biosensors are promising for point-of-care (POC) and home-diagnostics applications due to low cost and ease of miniaturization [6].

Most of the currently developed electrical biosensors use the faradaic process, where the charge is transferred across an interface and redox species are alternately oxidized and reduced by the transfer of an electron to and from the metal electrode (a redox process) [4,7,8]. Alternatively, a non-faradaic method has several advantages for POC applications [7]. The addition of redox species is not required in this case, and two electrodes are used instead of three. The need to add redox species makes the experiment more complicated because their properties and dosing accuracy also affect the measurement results. The use of the third (reference) electrode requires extra space in the sensor, and its properties (e.g., the stability) may reduce the accuracy of measurements significantly [9]. Moreover, the use of non-faradaic method allows shorter measurement time that provides better agreement with time invariance criterion of the system when monitoring time-dependent properties of the sample under test (SUT). The SUT is typically a small particle (e.g., cells, bacteria) in biocompatible solutions, e.g., in phosphate buffer solution (PBS).

The presented analyzer and sensor uses the non-faradaic method that allows a simpler design of small volume measurement cells with two electrodes, overwhelming the drawbacks of the faradaic method with the three electrode sensors discussed above.

The novelty of the proposed designs is related to the higher sensitivity of differential measurements with a capability to preserve actual impedance values, along with the design of the sensor with the 4-nanoliter volume of the measurement cell. The use of three direct and two differential measurement channels allows for sensitive enough comparison of properties of two SUT, with the third sample (the reference, which is typically clean PBS solution) used as a carrying medium for the first two channels.

The description of the impedance analyzer introduces the structure of measurement circuitry, focusing mainly on its analog front-end part. The used analog-to-digital (AD) and digital-to-analog (DA) converters, digital input-output (IO), and communication parts, are all described only briefly since a commercial digital oscilloscope module was in use for that purpose.

Several important topics related to the detection and measurement of small changes of electrical impedance spectra (EIS) in a fluidic environment are discussed as follows:

- Main measurement methods and typical shape of spectra
- Selection of excitation signal parameters (source type, frequency range, waveform, number of frequencies)
- Calibration procedure and related issues
- Short description of the software and user selectable parameters

In the discussion section, the results of the EIS measurements of the test set-up are analyzed. The introduced test analyzer is not the final solution for POC applications. The final versions will be certainly simpler, smaller, and cheaper. However, despite the moderate cost and dimensions, it provides flexibility and sensitivity, suiting experiments in the process of designing dedicated devices.

### 1.1. Impedance Measurement Methods and Excitation Signal Sources

To measure unknown impedance, we can apply a known voltage excitation across the object and measure the current flowing through it, or we inject a known current into that object and measure the voltage across. We can also measure both current and voltage simultaneously and calculate the impedance as their ratio. The last method is preferable if the excitation source does not generate a steady signal. This situation is typical for current sources at frequencies above 1 MHz, since their output impedance decreases because of stray capacitances and degradation of the performance of electronic components [3].

**Figure 1:**
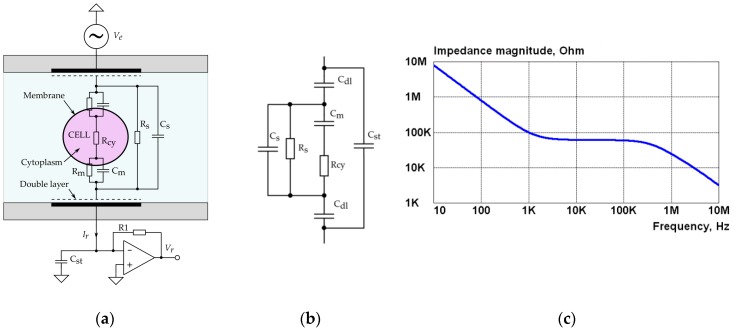
A magnitude spectrum (**c**) of the impedance of a single cell in saline solution (**a**) is described by a simplified electrical impedance model (**b**). The values of components are: C_dl_ = 4 nF, C_m_ = 1 pF, C_s_ = 2 pF, C_st_ = 3 pF, R_s_ = 60 kΩ, and R_cy_ = 100 kΩ. Magnitude spectrum (**c**) of the cell impedance is measured, applying an excitation voltage *V_e_* and measuring the response current *I_r_* through it. This is converted into a voltage *V_r_* using a transimpedance amplifier (TIA).

An example with a single cell in saline solution (Figure 1a) illustrates the formation of the impedance magnitude spectrum in the microfluidic sensor. The shape of the magnitude spectrum, measured by the aid of electrical impedance spectroscopy (EIS), typically has two decaying parts (Figure 1c). The first one is caused by so-called double layer capacitance that is formed near the surface of the electrode made of noble metals (e.g., gold or platinum). The second decaying part is introduced by the capacitance C_s_ of the liquid solution between the electrodes and by stray capacitances C_st_ formed from the input capacitance of both measurement electronics and the capacitance of connection leads and connectors. In the simplified electrical model presented in Figure 1b, the membrane resistance R_m_ is omitted, since its value is very high in comparison with solution resistance R_s_ and cytoplasm R_cy_. The capacitance C_dl_ is introduced in the simplified model (Figure 1b) instead of a constant phase element (CPE) used in more accurate electrical models discussed below. Note also that the detection of changes of R _cy_ and C_m_ would require high resolution since they are masked by significantly lower impedances of C_s_ and R _s_ connected in parallel. Typically, such design of sensors suits only for the characterization of mean values of a larger amount of cells. To address these issues, the detection area of the sensor should be small [10]. The novel design of the sensor (Section 2.2) provides a solution for that task.

In the case of using current excitation, the amplitude of the current must not produce a voltage drop above 50 mV on the double-layer impedance of electrodes to avoid significant nonlinearity of current-to-voltage relationship [3,4,11]. At higher frequencies, the use of a fixed (constant) current produces a low and decaying amplitude of the response voltage that decreases the signal to noise (SNR) of measurements. Note that in a higher frequency area (above 10 kHz in the current case) the voltage drop at the sensor is mostly determined by the resistance of the solution R_s_ and reactance of capacitances C_s_ and C_st_.

A constant voltage excitation *V_e_* (see Figure 1a) is better suited here, since the response current *I_r_* increases along with a decrease of the magnitude of the impedance. One more benefit of using the voltage source is that only the response current can be measured, since it is relatively easy to generate the stable voltage excitation in a required frequency range. Even if the measuring of excitation voltage is required for higher accuracy EIS measurement, the complexity of the voltage source is significantly less in comparison with a current source.

### 1.2. Required Frequency Range and Sensitivity

The required frequency range of EIS measurement depends on the properties of SUT. When investigating the properties of biological cells, it depends on the dielectric β-dispersion, which yields information on membrane capacitance, cytoplasm conductivity, and cytoplasm permittivity [3,4]. However, the required frequency range also depends on the dimensions of the measurement cell (chamber).

The cell constant *k*_g_ calculated as a ratio of the distance between the electrodes *l* and *a*. The surface area of the electrodes *A*, *k*_g_ = *l*/*A*, provide estimated values of R_s_ and C_s_ in the case when parallel plate electrodes also determine the dimension of the cell. If the more complicated shapes of the electrodes and cell are used, calculations of the cell constant become difficult. Therefore, the experimental determination of *k*_g_ using a test medium with known electrical properties and fixed environmental conditions is commonly used. The use of numerical simulations, such as the finite element method (FEM), allows one to predict and compare the influence of different electrode shapes.

**Figure 2:**
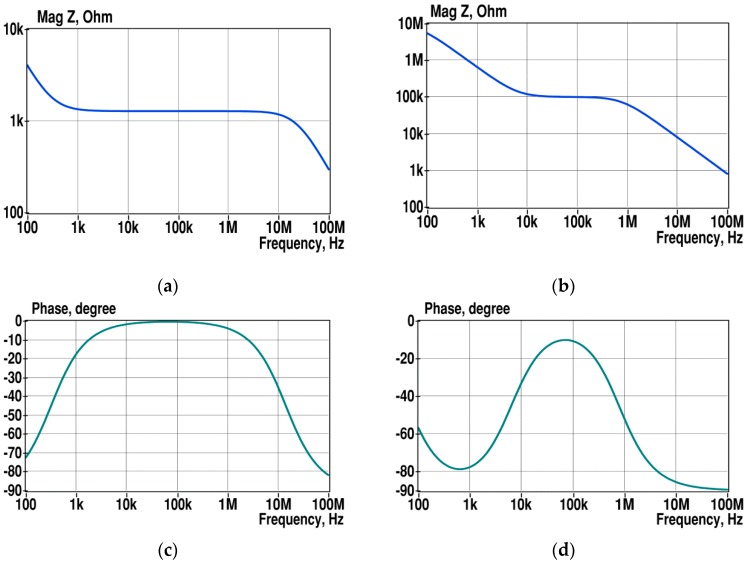
Typical spectra of the saline solution with a conductivity of 1 S/m with the following measurement cell parameters: *A* = 0.8 mm^2^, *l* = 1mm (**a**,**c**), and *A* = 0.0005 mm^2^, *l*_2_ = 0.05 mm (**b**,**d**). Double layer capacitances C _dl1_ = 400 nF, C _dl2_ = 250 pF, and the capacitances of the solution C_s1_ =13.8 pF, C_s2_ = 0.009 pF. The resistances of the solution R_s1_ = 1.25 kΩ, R_s2_ = 100 kΩ, and the stray capacitance C_st1_ = C_st2_ = 2 pF.

The sensitivity of detection of properties of small particles depends on the ratio of the volumes of particles and measurement cell and the placement of electrodes [10,11,12]. As shown in a previous study [13], the parallel plate electrodes provide better sensitivity and more uniform sensing area in comparison to other shapes. When the volume of the solution is higher than the volume of particles, the sensitivity of their detection decreases quickly, since the conductivity of the solution surrounding the particles is relatively high (typically 1 S/m in the case of biological objects). Decreasing the volume of the measurement cell, however, also decreases the surface area *A* of electrodes that influence the shape of the magnitude spectrum, as illustrated in Figure 2.

The smaller surface area of the electrodes accompanies a smaller capacitance of the double layer that shifts the first falling part of the magnitude spectrum toward higher frequencies. The second falling part of the spectrum is less influenced, since the contribution of stray capacitances C_st_ in overall parallel capacitance is relatively high. Shifting the first falling part of the EIS toward higher frequencies is not desirable for several reasons. At first, the β-dispersion area of SUT will depend more on the properties of electrodes, especially on C_dl_. Typically, the impedance of electrodes does not contain valuable information about SUT but adds variance into the total measurement result. Secondly, the SNR of EIS measurements decreases at higher frequencies, especially in the case of higher impedances. Moreover, when the conductivity changes are of interest, it becomes more complicated to detect them because of the strong influence of uninformative capacitive components (C_dl_, C_st_). In practice, the C _dl_ is not a pure capacitance, rather it is a CPE, which produces a large shift of the first part of EIS toward higher frequencies.

The effective surface area of the electrodes can be significantly increased by making them porous [14]. However, this solution leads to other problems (long settling time, contamination), as described in the discussion section. A large surface area of the sensor, presented here, is obtained with the novel spatial design of the electrodes.

In many practical situations, relative changes of the impedance of SUT are small in microfluidic sensors, which results in low SNR of the corresponding response signal. Typically, the amplitude of the response signal cannot be increased by lifting the amplitude of the excitation signal by its direct amplification, as this loses the system’s linearity [3,4].

A well-known solution for increasing both the sensitivity and resolution is a differential method. In this case, a reference signal compensates the basal value of the response signal, and the changes of the remaining signal are amplified, typically up to the second half of the full scale of the measuring device. The basal value may be obtained from the same dual microfluidic sensor, as shown in previous studies [4,11], from another (reference) sensor, or the results of modeling or previous measurements.

However, a fully differential measurement also has drawbacks and limitations. If only the differences in impedance spectra are measured, their actual (basal) value remains unknown. In the presented device, this problem is solved by combining both the direct and differential measurement methods, as described in the next section.

### 1.3. Selection of the Waveform for Excitation Signal and Number of Frequency Components

Different excitation signals are generally applicable in the wideband EIS. However, in electrical bioimpedance (EBI) measurements, some limitations set specific demands on the properties of the excitation signals [3,15]. As already noted, the amplitude of the excitation signal is limited to satisfy the criteria of linearity. Other limitations may appear when the investigation of living cells takes place in very small volumes. Even for non-biologic measurements, both the allowable input signal range and power supply voltage of electronic components limit the allowed amplitude of signals. Moreover, due to the high crest-factor (*CF*) of noisy signals, the increased probability of saturation of measurement channels leads to nonlinearities, which involve corresponding uncertainties in measurement results.

The selection of waveform for the excitation signal depends on the task, since the criteria for their efficiency are often contradictory, e.g., as are the SNR and speed of measurements. If the properties of SUT change quickly in time, the multifrequency waveforms, such as multisine and binary multifrequency signals (BMS) [15], are advantageous, since they may cover the frequency range of interest during the period of the lowest frequency component *f*_1_. However, this shortest measurement interval *T*_m_ = *T*_1_ = 1/*f*_1_ is at a cost of lower SNR in comparison with the measurement with a single sinewave with the same amplitude. In the case of fixed amplitude, the Root-Mean-Square (RMS) magnitudes of individual components in the multifrequency signals typically decay by $\sqrt{k}$ rule, where *k* is a number of frequency components. Moreover, the crest-factor (*CF*) above 1 also decreases the RMS value of all *i*-th frequency components of the multifrequency signal *S*_RMS_*(i)*

(1) $$S_{\mathrm{RMS}}{\left(i\right)}_{Max|s(t)|=1}=\frac{1}{\sqrt{k}\times CF}$$

The *CF* of the multisine signal depends on initial phases of its components. The multisine waveform with well-optimized phases may have a *CF* below that of a single sinewave ($\sqrt{2}$), but this applies mostly for consequent frequency distribution (*i* = 1, 2, 3, 4, *k*). The *CF* of BMS signal is always 1, which provides higher RMS values in the case of sparse frequency distributions [13], which is suitable for EBI measurements. Considering that the shape of the EBI spectrum is rather smooth, it is reasonable to spread the energy of the excitation only into a limited number of frequency components. The analysis shows that in certain cases using even three frequency components allows satisfactory fitting of values for the electrical model components [16].

Despite the BMS waveforms being the lowest, there are also disadvantages. Some part of the BMS energy always spreads onto higher harmonics of the desired frequency components. This part mirrors back onto lower frequencies and distorts the spectra (aliasing). Another important aspect is that the overall SNR of impedance measurements also depends on the CF of the response signal. The use of binary waveforms may significantly increase it in comparison with the sinusoidal waveforms [17]. In the presented analyzer all the excitation waveforms can be formed by the aid of an arbitrary waveform generator.

The stepped sinewave with multiple periods is preferred for getting better SNR results if the speed of impedance changes allows this.

## 2. The hardware of the Impedance Spectroscopy Analyzer

### 2.1. General Requirements

The objective of developing the EIS analyzer was to create a compact and inexpensive device with an integrated analog front-end, suitable for the realization of the measurement method described in the introduction. Modest energy consumption, good resolution of EIS measurements, a frequency range up to 10 MHz, and compatibility with sensors of different impedance ranges were also important criteria.

A compact analyzer for direct and differential EIS measurements with microfluidic sensors requires the following main parts and components:

- Connectors for short-distance connection of three microfluidic sensors intended for non-faradaic measurements
- Analog front-end part containing excitation signal source and response signal conditioning modules
- Analog-to-digital (AD) and digital-to-analog (DA) converters with logical input–output (IO) and data communication capabilities
- Software for controlling operating modes of the device, collecting and analyzing the measurement data, and presenting the analysis results
- Low noise power supply for the analog front-end, both external and integrated

### 2.2. Commercial Analyzers and Comparable Developments

There is a number of compact commercial instruments available. Most of these almost match the basic requirements, for example:

- MFIA Impedance Analyzer from Zürich Instruments AG, Zürich, Switzerland
- CompactStat from Ivium Technologies BV, Eindhoven, The Netherlands
- PalmSens4 from PalmSens BV, Houten, The Netherlands

Zürich Instruments MFIA is the most accurate, providing 16-bit resolution, 5 MHz bandwidth of analog input and output channels (including 10 µA to 10 mA current input ranges), and 60 Msa/s sample rate, but it is costly and not very compact (23.2 × 23.2 × 10.2 cm). CompactStats also has 16-bit resolution, its dimensions are 12 × 26 × 25 cm, but the bandwidth of analog input–output is limited to 3 MHz. PalmSens4 is the smallest in this list, at 15.7 × 9.7 × 3.5 cm. However, it is dedicated mostly to Potentiostat and Galvanostat measurements, and its EIS bandwidth is 1 MHz.

Many developments of compact and inexpensive EIS analyzers have also been carried out during the last decade; some of them are discussed in a review [18], and the most compatible are described in previous studies [19,20,21].

The portable EIS analyzer introduced in a previous study [19] uses the STEMlab 125-14 module from Red Pitaya d.d., Solkan, Slovenia with two channel 14-bit 125 MSa/s AD and DA converters, ARM processor, field-programmable gate arrays (FPGA), and interface devices. The software for the electrical impedance tomography (EIT) application is written in Matlab to control the FPGA and processor, collect the data, and analyze the results. The dimensions of the STEMlab module without enclosure are 107 × 60 × 21 mm, but the developed systems also consist of Analog Multiplexer and Voltage Controlled Current Source modules. Its EIT system works in the frequency range from 1 kHz to 100 kHz. The overall power consumption is not provided, however, the STEMlab itself consumes 10 W (2A from 5V).

The compact FPGA-Based Elaboration Platform for wide-bandwidth electrochemical measurements [20] covers a frequency range up to 10 MHz. A 14-bit DA converter for the excitation signal generation and two 14-bit AD converters both work with 80 MSa/s sample rate. The output voltage range for EIS measurements is ± 0.5 V and the input ranges are ± 2 and ± 10 V. The dimensions of the module are 15 × 24 cm, however, the transimpedance amplifier (TIA) required for the impedance measurement is external. Current consumption of the module is 700 mA from ± 12V. Measurement of the dummy cell with R _ct_ = 1 MΩ in parallel with C _dl_ = 56 pF and R _s_ = 10 kΩ gave a 5 % error for magnitude and 1° for the phase in the 10 MHz bandwidth.

The Data Acquisition (DAQ) module-based system [21] was declared as a low-cost and portable EIS measurement system, which uses National Instrument’s 24-bit USB-4431 DAQ board with an additional amplifier board. Considering the frequency range (from 100 Hz to 10 kHz) and also the dimensions and cost of the DAQ module (over 3000 USD), this version does not suit the current task.

An important common drawback of both commercial instruments and dedicated developments is that they do not provide integration of the required analog front-end part.

### 2.3. General Composition of the Analyzer and Novel Sensor

The analyzer covers the frequency range from 0 to 10 MHz. A voltage source is used for excitation to avoid limitations of current sources described in the introductory section. However, because the voltage source is not ideal, we measure both the response current *Ir* and the excitation voltage *Ve* simultaneously to get better accuracy. The complex impedance spectrum $\vec{Z}(f)$ of SUT is calculated using a Fourier transform ℱ:

(2) $$\vec{Z}(f)=ℱ(V_{e}(t))/ℱ(I_{r}(t))$$

**Figure 3:**
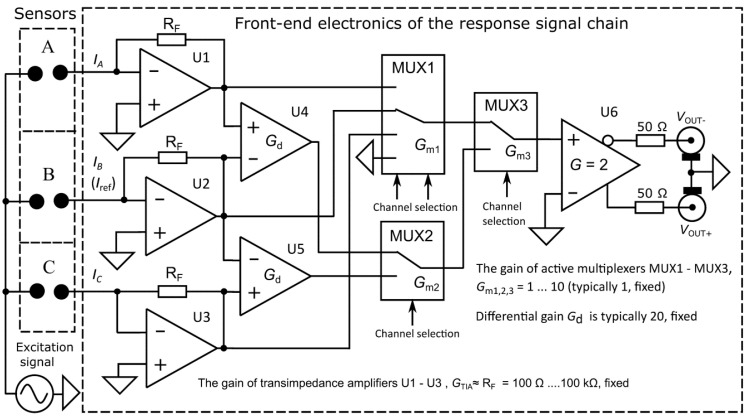
Structure of the front-end electronics of the response module. The signal of channel B is used for acquiring the impedance differences with sensors A and C. The signal differences from the outputs of U1 and U2, and, U3 and U2, are amplified by amplifiers Gd. Both direct and amplified differential signals of all channels are forwarded to the output via high-speed multiplexers MUX1 and MUX2, allowing calculation of actual values of amplified differences of impedances. The reference signal of channel B may be obtained from a sensor connected to it, or from a reference signal source.

Using the differential method increases the sensitivity and resolution of measurements. The basal value of the response signal is compensated, and the remaining small signal changes are amplified. The developed analyzer allows restoration of actual impedance values from the results of differential measurements by combining both direct and differential methods, as illustrated in Figure 3.

Despite the better sensitivity of the differential method, the improvement of the SNR of measurements remains typically modest, since the overall SNR depends significantly on the properties of the first amplifier stages in the response signal chain (transimpedance amplifiers, TIA, in our case), and the internal noises of the sensors. Nevertheless, the simultaneous employment of several sensors allows suppression of common disturbing factors (e.g., temperature changes, common electrical interferences), which significantly increases the measurement accuracy. Moreover, the distortions of the common excitation signal are also cancelled out, at least partially. Other important factors influencing the overall efficiency are the selection of optimal excitation signals discussed in the introductory section, signal processing methods, and presentation of measurement results.

### 2.4. Microfluidic Sensors

**Figure 4:**
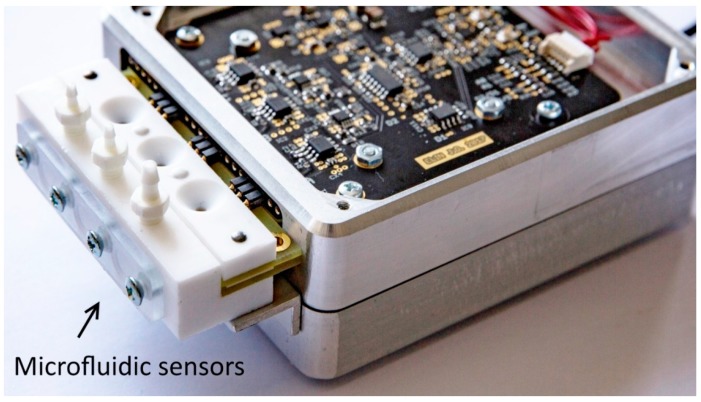
Analyzer with the 3-channel sensor exposing the response chain’s module of the front-end (top cover removed). The PCB of the excitation module is fixed directly underneath the response module’s PCB. Signal processing module AD2 with AD and DA converters, logical IO, and communication capabilities is situated in a lower part of the aluminum case, separated with a partitioning wall for shielding.

Microfluidic sensors are to be connected directly with analog front-end connectors, as illustrated in Figure 4. The shortest connections provide minimal stray inductances and capacitances that ensure minimal distortion of the impedance spectra, lower noises of the input TIA stages [11], and lesser susceptibility to external disturbances.

The use of several sensors with identical properties in the same compartment, preferably, is essential for the differential method. At first, this allows easy separation of the changing part of properties from their basal (reference) values. Secondly, the identical and united design of sensors allows a significant reduction of the influence of common disturbing factors.

**Figure 5:**
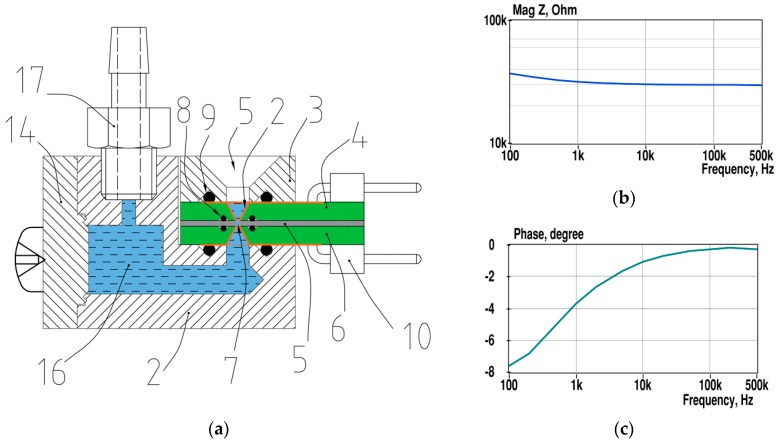
Cross section view (**a**) of the sensor adapted from patent application (given in the patent section) and the measured spectra (**b**,**c**) of the PBS solution with a conductivity of 1.3 S/m. Gold-plated electrodes (2) are formed by two PCBs (4 and 6), and a miniature measurement chamber (7) with a thin dielectric layer (5.) The height of the conical electrodes is 1.6 mm, and the diameter of their base is 0.8 mm. The height of the measurement chamber is 0.5 mm and its diameter is 0.1 mm.

The introduced novel design of the microfluidic sensor provides a miniature measurement chamber with increased surface area of electrodes. A significant increase of the surface area is achieved with the spatial, preferably conical shape of the electrodes (Figure 5a). This shape also reduces turbulences when fluid is flowing in the fluidic channel.

The liquid is inserted into the funnel-shaped opening (5), and can be guided between the electrodes (2) of the measuring chamber (7) using an under-pressure through the nipple, but also by overpressure through the nipple and reservoir (6). By changing the pressure direction, the fluid moves in both directions, for mixing, as an example. The electrode connections run into the connector (10). Pressure resistance is achieved with the sealing O-rings (8 and 9). The volume of the measurement chamber is around 4 nanolitres and the surface area of electrodes is around 2 mm^2^. That provides the almost flat shape of the impedance magnitude curve starting at 1 kHz (Figure 1b). The phase deviates only 4 degrees from zero at this point.

Other details of the sensor are made of chemically inert plastic, e.g., Teflon, and the openings (5) have an additional cover for avoiding evaporation. Note that the design of sensors presented here is an application example that may be modified in several ways, as described in the patent application P201900007. For example, the fluid channel may be composed of two horizontal halves. The important aspect is that the cross-section of pairs of similar spatial electrodes decreases smoothly towards the tiny measuring chamber to increase the sensitivity and accuracy of the measurement.

The EIS measurements were conducted with phosphate buffer solution (PBS), 137 mM NaCl, 2.7 mM KCl, and 10 mM phosphate using one tablet of Sigma-Aldrich (St. Louis, MO, USA)# 79382-50TAB dissolved in 200 mL distilled water. Distilled water was obtained from Sartorius arium^®^ mini Ultrapure Water System (Sartorius Lab Instruments GmbH & Co. KG, Goettingen, Germany).

### 2.5. Response Signal Module

The task of the analog front-end is conditioning of excitation and response signals by division, amplification, conversion, multiplexing, and impedance matching. The structure of the response signals chain is shown in Figure 3. Currents from all the sensors are converted to voltages by ADA4817 transimpedance amplifiers (TIA) U1, U2, and U3, and forwarded via AD8174 and AD8170 based multiplexers MUX1 to MUX3 to the output stage U6, which employs an ADA4950 amplifier with differential outputs matched to 50 Ω coaxial cables. Amplified differences of A-B and C-B from the outputs of TIAs go further simultaneously through differential amplifiers U4 and U5. (AD8129) All the given integrated circuits are from Analog Devices (Analog Devices Inc., Norwood, MA, USA). Assuming that the amplitude of the excitation voltage is fixed (typically at the level of ± 50 mV), the required gains of TIAs and differential amplifiers depend on the impedances of sensors and distortion-free range of output voltages (typically ± 3 V). Matching with the input range ± 1 V of AD-converters is also important. The signal amplitude at the output stage (see U6 in Figure 3) must be below ± 2 V, considering that the resistors used for matching the impedance 50 Ω of coaxial cables produces a division of signals by 2.

The gain of TIA stages U1–U3 is set with resistors R_F_ ranging from 1 kΩ to 100 kΩ. The required gain depends on impedance ranges of SUT. For the better SNR of the following AD-converters, it is desirable to keep the signal level near their full scale. However, the choice of R_F_ value also influences the noise and bandwidth properties of TIA [11], which limits the overall performance of the EIS analyzer. For the full range of frequencies (10 MHz), the R_F_ value should not exceed 10 kΩ. The additional gain is obtainable from buffered multiplexers MUX1–MUX3. Selected multiplexers provide fast and accurate switching capabilities (15 ns to 0.1% level) and low distortions (below –60 dB at 10 MHz). In the current analyzer, all the gains are set with 0.1 % precision fixed resistors.

### 2.6. Excitation Signal Module

**Figure 6:**
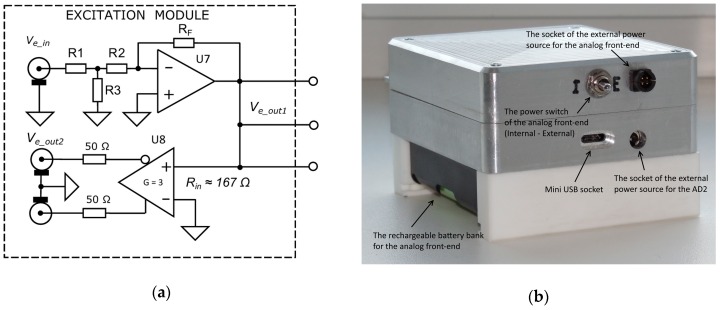
Structure of the front-end electronics of the excitation module (**a**) and backside of the analyzer with dual polarity 3 Ah rechargeable battery bank at the bottom (**b**).

The structure of the excitation signal module is shown in Figure 6, in which a ± 1 V full-scale input signal *V_e_in_* comes from an arbitrary waveform generator (AWG) in the signal processing module. A T-divider on resistors R1, R2, and R3 divides the signal by 10 and matches with the impedance of the 50 Ω coaxial cable. The feedback resistor R_F_, in conjunction with divider’s resistance and 50 Ω output resistance of the excitation signal source, defines the overall gain of the stage employing an ADA4899 driver amplifier U7 with low output impedance, low harmonic distortions, and low offset voltage. The output signal of the driver ((amplitude ± 50 mV) is applied to all sensors and the input of the excitation voltage measurement chain with a buffer-amplifier stage U8 with differential outputs matched to 50 Ω coaxial cables. This part uses the same ADA4950 low power amplifier with with high speed, low power and low harmonic distortions (below −100 dB at 10 MHz), operating as the output in the response signal module. The gain is set to 3 with on-chip resistors that are also defining input resistance near 167 Ω. This input resistance is sufficient when considering low output impedance (0.6 Ω at 10 MHz) of the driver and synchronous measurement of the excitation voltage for impedance calculations according to (2).

### 2.7. Power Supply and Physical Dimensions

Both analog front-end modules are powered by a dual polarity rechargeable battery bank with an output voltage range from ± 6 V to ± 8 V or from an external adapter. The use of batteries is preferred for galvanic isolation of the sensitive analog front-end part to avoid disturbances from the mains ground loop. Current consumption is 120 mA ± 10%. The input amplifiers (TIA) of the response module and the rest stages of it use separate linear voltage regulators that improve decoupling of the power supply of circuit sections and distributes the power losses. ADP7118ACPZ is used for + 5V and ADP7182ACPZ for – 5V, both from Analog Devices (Analog Devices Inc., Norwood, MA, USA). These regulators provide high-level wideband power supply rejection ratio (PSRR), low noise, and excellent line and load transient response with a small 2.2 µF ceramic output capacitor. The excitation module is equipped with voltage regulators of the same types.

The signal processing module AD2 is powered from the USB port of the PC and consumes 350 mA ± 10%. In cases where large sensor units are used, or the sensors unit is not directly placed near the front-end electronics connector, the USB port should be galvanically isolated using fibre optic devices, such as LINDY’s USB 2.0 MM Fibre Optic Extender, Part No. 42702 from LINDY International Limited, Thornaby, UK.

The prototype of the analyzer has dimensions of 90 × 125 × 40 mm (without batteries). With a 3 Ah battery bank at the bottom, the hight increases to 63 mm (Figure 6b).

### 2.8. Signal Processing Module

The use of two-channel synchronous high resolution and high-speed AD and DA converters is a common solution for EIS measurements. In the current case, digital IO channels were also required to control the front-end multiplexers. The analog frequency range up to 10 MHz, data acquisition via a standard interface, modest energy consumption, and small dimensions were important criteria. There are several commercial products available that mostly meet these requirements, e.g., Red Pitaya STEMlab 125-14 module employed in a previous study [19] and USB oscilloscope Analog Discovery 2 module (later here AD2) from Digilent Inc., Pullman, WA, USA. Our choice was AD2, mainly because of its significantly lower supply power (0.35 A, 5V against 2A, 5V) and smaller dimensions. The signal processing module with lower energy consumption generates less heat, which makes it better suited for integration with analog front-end and sensors that are sensitive to temperature changes.

**Figure 7:**
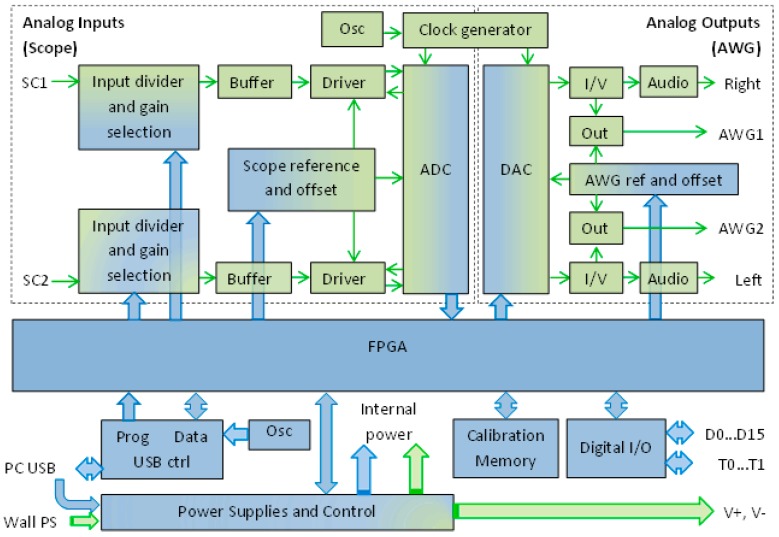
Structure of the Analog Discovery 2 (AD2) module adapted from its datasheet.

The structure of the AD2 module is shown in Figure 7. The AD2 provides two DA converters with arbitrary waveform generation (AWG) capability. The AD2 provides two synchronous 14-bit 100 Msample/s AD-converters and DA converters with arbitrary waveform generation (AWG) capability. The input voltage ranges of AD converters are ± 1 V and ± 10V, and the output voltage range of the AWG outputs is ± 1 V. The high input impedance (1 MΩ with 24 pF in parallel) of AD channels is reduced near to 50 Ω with external resistors to ensure less susceptibility to external disturbances.

One of the AWG outputs is used for the excitation signal, and another may be used for the creation of the input signal for the reference channel discussed in the introduction section. The excitation signal Ve_out2 from the excitation module (Figure 6a) and the response signal from the response module (Figure 3) are both digitized synchronously by two A-D converters. The selection of sensor channels and their operating mode (direct or differential) is accomplished by multiplexers, controlled by digital IO outputs of the AD2 module.

A USB2 type controller of AD2 interfaces with the PC to program the volatile FPGA memory after settling of power-on state, or a new configuration is requested. After that, it performs the data transfer between the PC and FPGA. In our application, the AD2 module is powered from the USB port of the PC or Fibre Optic Extender.

**Figure A1:**
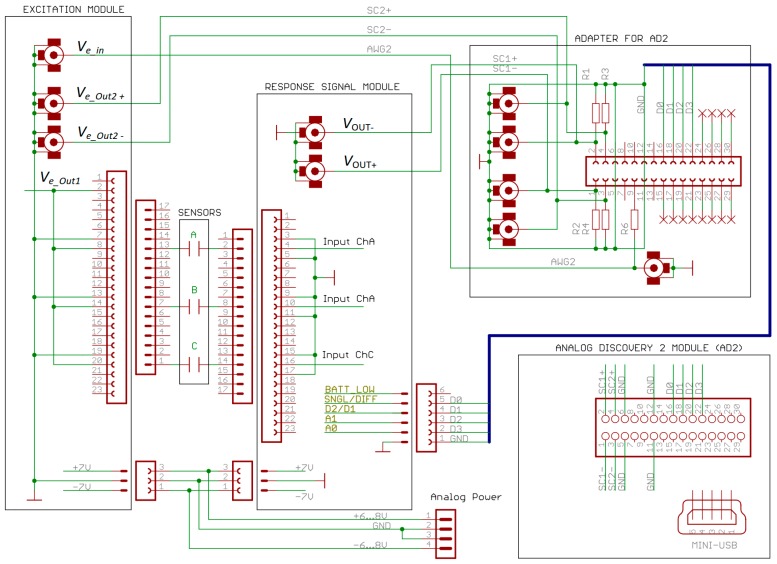
Connection diagram of analyzer modules.

**Figure A2:**
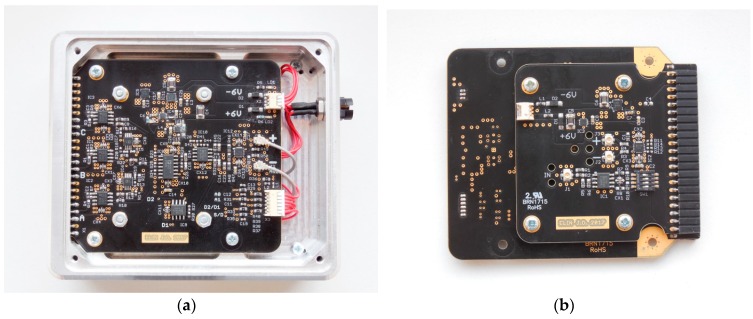
A photograph of the response chain’s module of the front-end (**a**) (top cover of the analyzer is removed) and the excitation module (**b**) of the front-end, fixed underneath of the same response chain module. Note: the power supply switch shown in Figure 6 is not implemented here.

**Figure A3:**
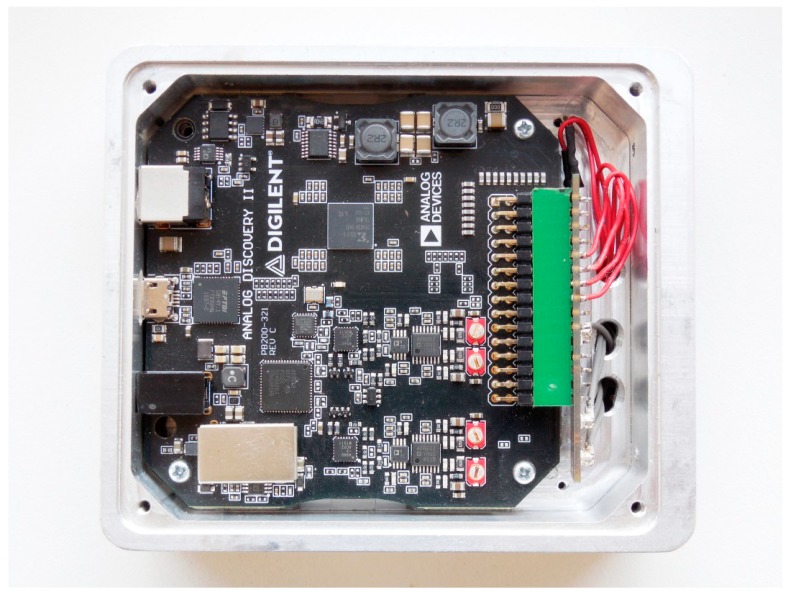
A photograph of the signal processing module with Analog Discovery 2 (bottom cover of the analyzer is removed).

Additional photos of analyzer parts and information about their connections are provided in Figure A1, Figure A2 and Figure A3, Appendix A.

## 3. Software and Signal Processing

### 3.1. Software

The PC-type host computer controls the analyzer settings, signal processing, and graphical data representation of measurement results using the developed software running in National Instrument’s LabVIEW programming environment. The host computer and LabVIEW perform the Fourier transform of the collected signals. In comparison with the FPGA-based solution, this allows lower complexity and energy consumption of the signal processing module. Control of the AD2 signal processing module (see Figure 7) is performed using the free WaveForms software development kit (SDK) provided by Digilent, which also contains virtual instrument files (VI-s) for LabVIEW. The setting of operating modes, the signal processing parameters, and data collection modes together with monitoring of running measurement results is performed via a graphical user interface (GUI) of the MAIN software. The results of EIS measurements are recorded into text files. The recorded data is used later for extra data processing and visualization with additional software, also developed in the LabVIEW environment. The MAIN program provides control of all operating modes and parameters, including:

- Parameters of the excitation signal (waveform, list of frequencies, amplitude, number of signal periods in one record, number of signal points)
- Parameters of input channels (full scale, sampling frequency)
- List of input channels and their sequence (e.g., A, B, “A-B”, C, “C-B”)
- Channel timing parameters (delay between switching events, delay after switching)
- Recording length (with timer)

Monitoring and visualization of running measurement results include:

- Waveforms of the excitation and response signals
- Spectra of the EIS magnitude and phase
- Change of the magnitude and phase against the previously recorded value
- Normalized magnitude spectra of the excitation and response signals (optional, for monitoring of spectral purity)

A file name of the recorded data contains a short label of the experiment, date, and time. The data in the text form is organized as follows:

- Comment rows that are filled with additional data about conditions of the experiment
- A row with a list of excitation frequencies
- Pairs of following data rows containing the running time, magnitude, and phase information

The recorded data is used later with developed extra signal processing and visualization software that allows different presentations and additional processing:

- Mean values of collected spectra during the selected time interval
- Spectra in a magnitude/phase and real/imaginary part (Nyquist plot) presentation
- Additional filtering of spectra
- Differences in spectra of various experiments
- Variation of spectra during selected time interval
- Changes of spectra in time at a selected frequency

### 3.2. Initial Calibration

Several calibration procedures are to be carried out for the improvement of accuracy and repeatability of measurements. For the initial calibration step, fixed precision resistors are connected in place of microfluidic sensors to calibrate the whole signal chain of all sensor channels. The nominal values of resistors match the mean value of expected resistance of sensors in the center part of the frequency range.

The absolute accuracy of EIS measurements provided by such a simple calibration procedure may significantly decrease at the high-frequency end (above 2 MHz), as discussed in the results and discussion section. However, for comparative measurements, it is still a satisfactory solution.

**Figure 8:**
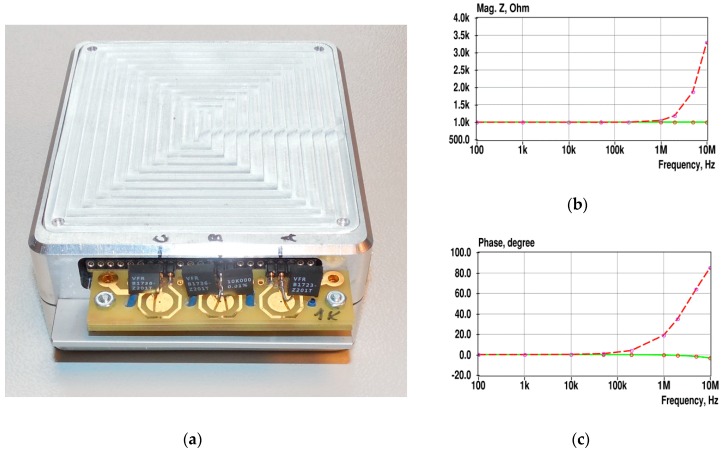
Precision resistors connected into a place of sensors for calibration (**a**), and the spectra (**b**,**c**) of the impedance of the 1 kΩ resistor connected to channel A before (dashed lines) and after usage of initial correction coefficients (solid lines). Ring-shaped marks indicate the used frequencies.

After collection of the measurement data, the correction coefficients $\vec{K}(i)$ are calculated for all used frequencies from *f*_1_ to *f*_k_ as follows:

(3) $$\vec{K}(i)={\vec{Z}}_{r}(i)/{\vec{Z}}_{m}\left(i\right),$$

where *i* = 1, …, *k*, and ${\vec{Z}}_{r}\;\mathrm{and}\;{\vec{Z}}_{m}$ are the impedances of the precision resistors and measured impedances, respectively. The correction coefficients are calculated for all sensor channels for their later use as $\vec{Z}(i)=\vec{K}(i)\times {\vec{Z}}_{m}\left(i\right)$. Note that the correction coefficients and impedances are given here in a complex form. For graphical representation, the impedance spectra are usually depicted as magnitude and phase spectra or Nyquist plots. In Figure 8b, an example of the magnitude and phase spectra of the impedance of a 1 kΩ resistor connected to channel A before and after the use of initial correction is shown.

### 3.3. The impedance of Differential Channels

Initial calibration of differential channels “A-B” and “C-B” (see Figure 3) is similar to initial calibration of direct channels described in the previous section, but the value of the calibration resistor in channel B is selected so that it provides small known differences, e.g., ± 10%, in comparison with resistances of channels A and C.

The actual values of impedance differences $d\vec{Z}*$ of sensor channels “A-B” and “C-B” can be calculated for each frequency *f* (*i*) as

(4) $$d\vec{Z}{*}_{AB}(i)=\frac{{\vec{Z}}_{A}(i)\times {\vec{Z}}_{B}(i)}{{\vec{Z}}_{AB}(i)};\;d\vec{Z}{*}_{CB}(i)=\frac{{\vec{Z}}_{C}(i)\times {\vec{Z}}_{B}(i)}{{\vec{Z}}_{CB}(i)},$$

where ${\vec{Z}}_{A},\;{\vec{Z}}_{B},\;{\vec{Z}}_{C}$ are the impedances, obtained from direct channels, and ${\vec{Z}}_{AB},\;{\vec{Z}}_{CB}$ are from differential channels. Note that the calibration correction coefficients should be found for both signs (+/-) of the difference signal, and their later selection depends on the sign of the difference of impedance magnitudes obtained from direct channels. This method also assumes that the speed of changes of the impedances of SUT is at least one order slower in comparison with a duration of the measurement cycle of all channels.

## 4. Results and Discussion

The EIS measurements were conducted with three different sensor types using the PBS solution described at the end of Section 2.4 with a conductivity of 1.3 S/m. The temperature of the laboratory environment was 23 ± 2 °C and relative humidity was 50 ± 10%. In all the experiments, the following parameters of the measurement setup are taken, if not stated otherwise:

- Stepped sinewave voltage excitation with an amplitude of 50 mV
- The delay for settling signals after commutation of channels is 10 ms
- The number of collected signal periods is 80
- The delay for settling signals after a change of the frequency is ten signal periods
- The sampling rate of AD and DA converters is 100 Msa/s
- The input range of AD2 module is ± 1 V
- Other measurement parameters are described additionally.

### 4.1. Measurement Results with a Dummy Cell

The impedance of “Dummy Cells” as SUTs was measured with both the developed analyzer and the commercial precision impedance analyzer Wayne-Kerr 6500B (Wayne Kerr Electronics, Bognor Regis, UK), to validate the correct operation of the new measurement device. The first SUT is formed as a parallel connection of 2 kΩ resistor and 10 pF capacitor in series with a 10 nF capacitor. The second SUT contains the same capacitors and a 10 kΩ resistor. Both resistors show 0.01% tolerance, low inductance (< 80 nH), and 2 ppm/°C temperature coefficient. The tolerances of silver mica type 10 nF capacitor and of 10 pF NP0 type ceramic capacitor are ± 5%. Initial calibration of the developed analyzer performed with 2 kΩ precision resistor is as described in Section 3.2.

The relative error of the impedance magnitude and difference in phases is obtained by comparison of the mean values of collected results over 5 min. The excitation voltage amplitude was ± 50 mV for both instruments.

**Figure A4:**
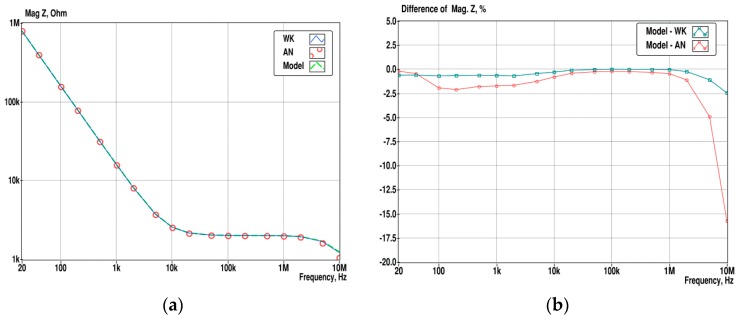
Impedance magnitudes (**a**) of the SUT1 measured with Wayne Kerr analyzer (WK), the developed analyzer (AN), and calculated from the computer model with the nominal values of its components; (**b**) relative deviations of the model’s impedance magnitude from the values measured with WK and AN.

**Figure A5:**
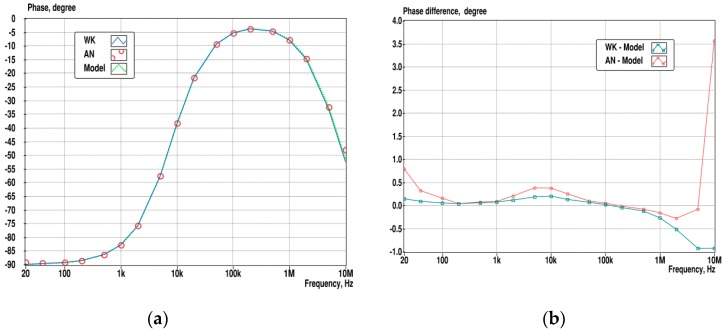
Impedance phases (**a**) of the SUT1, measured with the Wayne Kerr analyzer (WK), the developed analyzer (AN), and calculated from a computer model at the nominal values of its components. Deviations of phase values (**b**), measured with WK and AN, from the modeled results.

**Figure A6:**
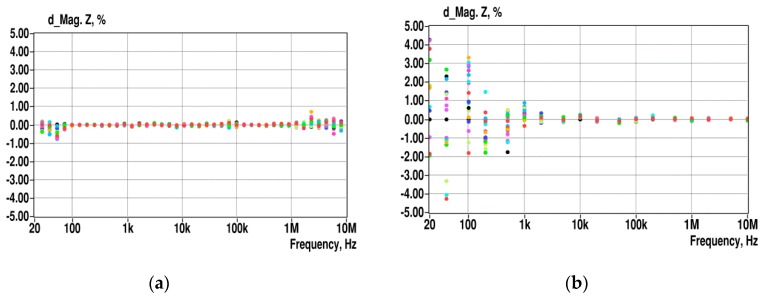
Impedance magnitude variations of the SUT1 measured with the Wayne Kerr analyzer (**a**), and the developed analyzer (**b**).

**Figure A7:**
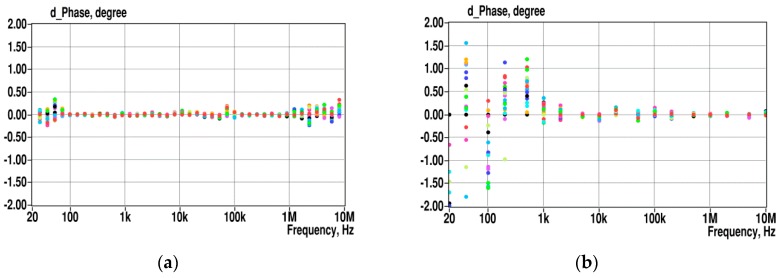
Impedance phase variations of the SUT1 measured with the Wayne Kerr analyzer (**a**) and the developed analyzer (**b**).

**Figure A8:**
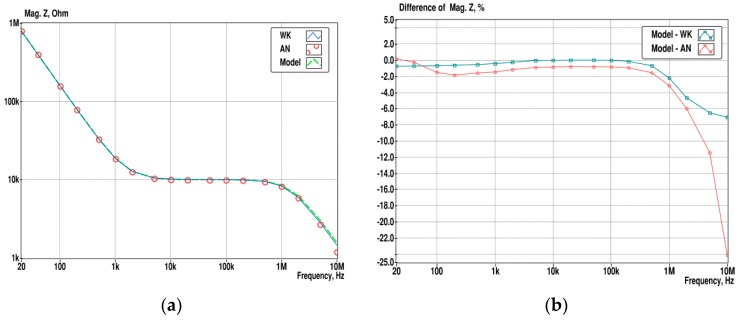
Impedance magnitudes (**a**) of the SUT2 measured with the Wayne Kerr analyzer (WK), the developed analyzer (AN), and calculated from the computer model with the nominal values of its components; (**b**) relative deviations of the model’s impedance magnitude from the values measured with WK and AN.

**Figure A9:**
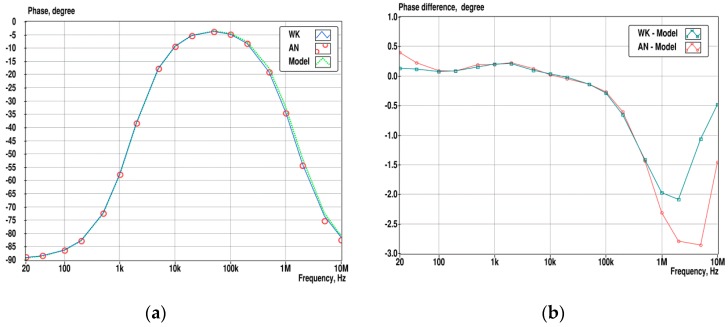
Impedance phases (**a**) of the SUT2, measured with the Wayne Kerr analyzer (WK), the developed analyzer (AN), and calculated from a computer model at the nominal values of its components. Deviations of phase values (**b**), measured with WK and AN, from the modeled results.

**Figure A10:**
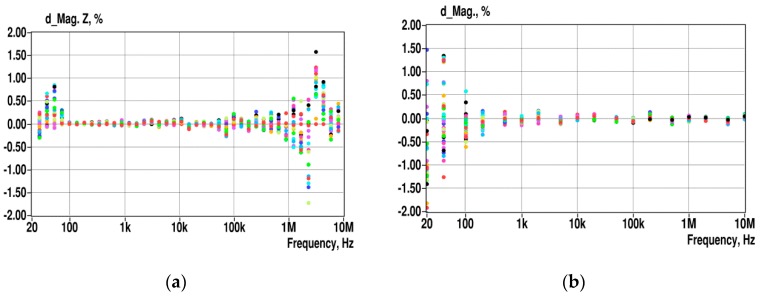
Impedance magnitude variations of the SUT2 measured with Wayne Kerr analyzer (**a**) and the developed analyzer (**b**).

**Figure A11:**
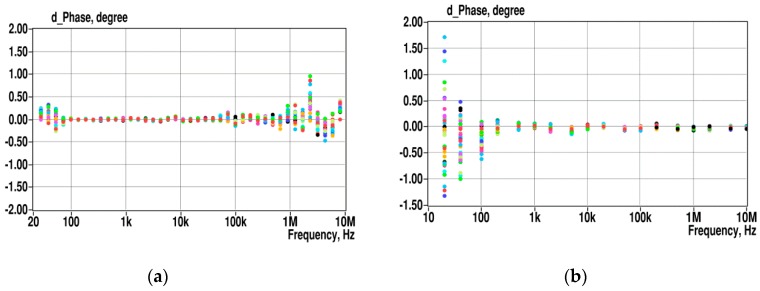
Impedance phase variations of the SUT2 measured with the Wayne Kerr analyzer (**a**) and the developed analyzer (**b**).

The relative error of the impedance magnitudes in the frequency range from 20 Hz to 2 MHz is below 2% and the phase errors are below 1°, but increase up to 22.5% and 4.5° at the higher end frequency of 10 MHz. More detailed information about the comparison of measurement errors is provided in Appendix B, Figure A4, Figure A5, Figure A6, Figure A7, Figure A8, Figure A9, Figure A10 and Figure A11, and the sources of uncertainty are discussed in Section 4.4.

Despite the lower accuracy when measuring the absolute values of impedances, the proposed analyzer is still well-suited for its main and direct purpose—the detection of relative changes of the impedance in the β-dispersion area. This is proved by the results provided in the following sections discussing the measurements with different fluidic sensors. Moreover, the comparison with the precision commercial analyzer Wayne-Kerr 6500B shows similar or even lesser deviations of the impedance values in the range from 10 kHz up to 10 MHz (see the comparison in Figure A6, Figure A7, Figure A10, Figure A11, Appendix B).

### 4.2. Measurement Results with a Novel Sensor

**Figure 9:**
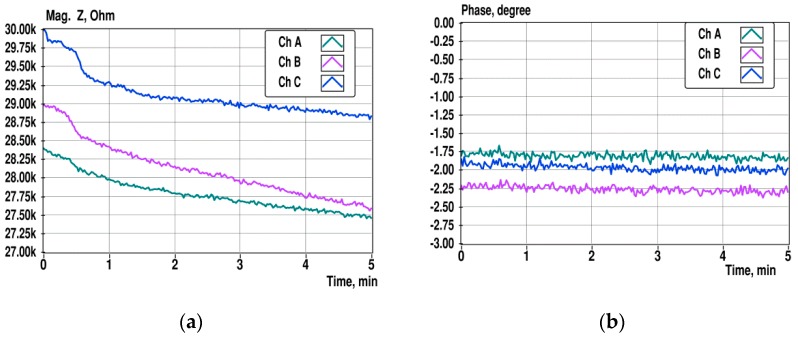
Changes of the impedance magnitude (**a**) and phase (**b**) at 10 kHz during 5 min observation in sensor channels A, B, and C.

In Figure 9, the variations of the impedance spectra in time at a single frequency of 10 kHz are shown for the sensor channels A, B, and C. The excitation has seven frequencies ranging from 1 kHz to 100 kHz, and the data of 225 impedance measurements were collected with 1.36-s intervals during 5 min.

Changes in the impedance magnitude over 5 min are ranging of 3–5%, and the difference of initial values is up to 5%. The changes of the impedance magnitudes may be caused by the temperature dependence of the solution’s conductivity, which is around 2%/degree [22], and the differences of initial values is probably caused by differences of dimensions of measurement cells. However, it is clearly noticeable that the slopes of magnitude changes of different channels are not the same. This situation makes it difficult to measure the small differences in impedance.

**Figure 10:**
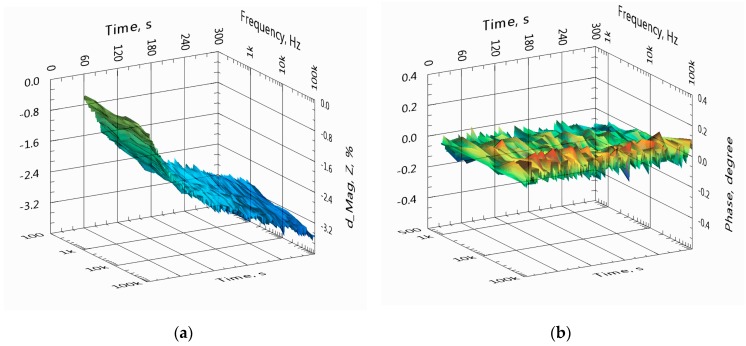
Overall variation of the impedance magnitude (**a**) and phase (**b**) of spectra over 5 min. The spectra were collected from the same sensor and at the same timeframe as presented in Figure 9.

In Figure 10, the overall relative variation of the impedance spectra of channel A at seven frequencies is shown in the same timeframe as presented in Figure 9.

**Figure 11:**
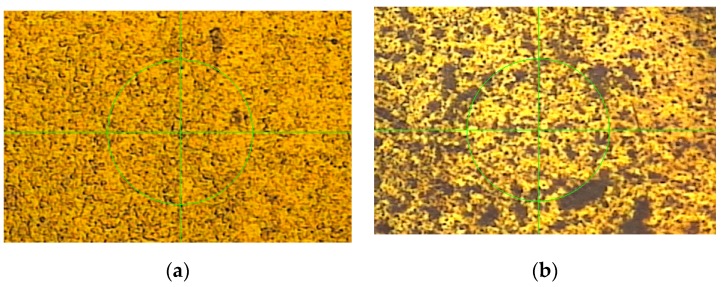
Images of surfaces of the PCB electrode (**a**) and AC2P electrode (**b**) discussed in the next section. The diameter of the green cursor circle is 150 µm.

Further experiments with other sensors discussed in the next sections show that the main reason for the impedance divergence is the porosity of electrode surfaces of experimental sensors (Figure 4 and Figure 5). The porosity is caused by the PCB technology used (Figure 11a). The images are obtained with a Visio^®^ 200-GL optoelectronic measuring machine from Tesa SA, (Renens, Switzerland).

**Figure 12:**
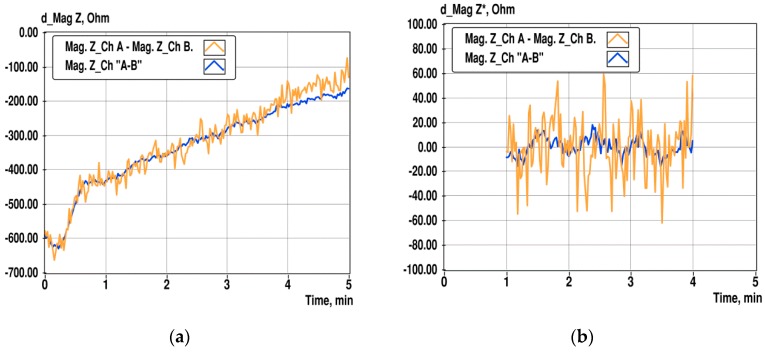
Change of the difference of impedance magnitudes in channels A and B at 10 kHz, and in the differential channel “A-B” during 5 min of observation (**a**), and linearized differences of the same data in the time interval from 1 to 4 min (**b**).

Despite the long settling time and divergence process of the impedance that is caused by the sponge-like structure of the gold-plated PCB surface, the proposed differential method still provides significant improvement of the SNR of the impedance measurement, as illustrated in Figure 12. For the comparison of results obtained by subtraction of impedance magnitudes of direct measurement channels A and B, (d_Mag Z), and from differential channel “A-B”, d_Mag Z*, as a magnitude of the complex impedance calculated according to Equation (4). The middle part of the data in Figure 12a is straightened using a linear fit with the least-squares method.

Calculated standard deviation (STD) of d_Mag Z is 23.5 Ω and 7.3 Ω for d_Mag Z*. Corresponding variances are 552 and 53.6 respectively. It may be concluded that the SNR of difference measurement improves around ten times. Relative standard deviation (RSD) of d_Mag Z against the mean impedance magnitude in channel A is 0.09%, and 0.03% for d_Mag Z* obtained from differential channel measurement.

### 4.3. Measurement Results with AC2P Sensor

**Figure 13:**
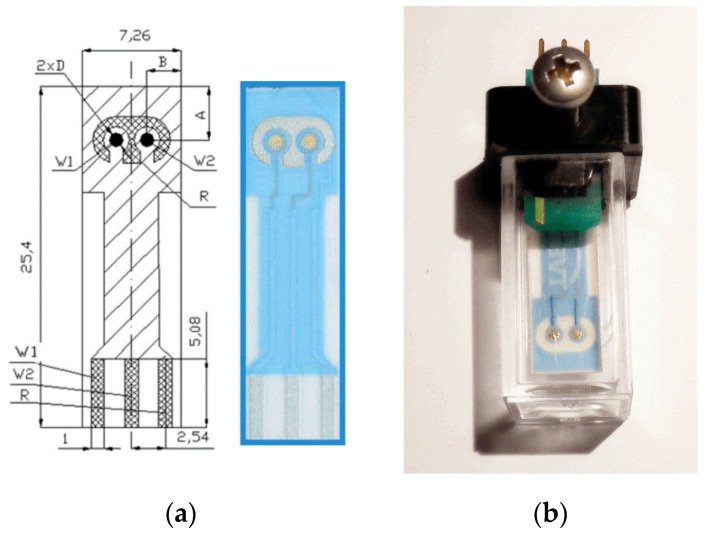
Images of the original AC2P.W1 sensor (**a**) and the modified sensor in a cuvette (**b**). The diameter of the spot electrodes is 1 mm, and the distance between their centers is 2.5 mm.

To investigate the influence of the electrode surface porosity on impedance settling process, an experiment with the AC2P.W1 sensor from BVT Technologies s.a., (Strážek, Czech Republic) (Figure 13) was carried out. The third silver plated electrode was removed with nitric acid, and two remaining gold-plated electrodes were connected to channel A of the EIS analyzer with the adapter fixed on a top of the rectangular cuvette containing 1 mL of PBS solution.

Though the surface electrodes of the ACP2 sensor also appears to be not very flat (see Figure 11b), it provides a significantly lesser settling deviation of the impedance magnitude in comparison with the spongy structure of the gold-plated PCB surface, as shown in Figure 13. A rectangular moving average filtration with a half-width of 8 samples is used here additionally to suppress high-speed fluctuations.

Stepped sinewave excitation has nine frequencies ranging from 100 Hz to 10 MHz. The data of 212 impedance measurements were collected with a near to 1.4-s interval over 5 min in this experiment.

**Figure 14:**
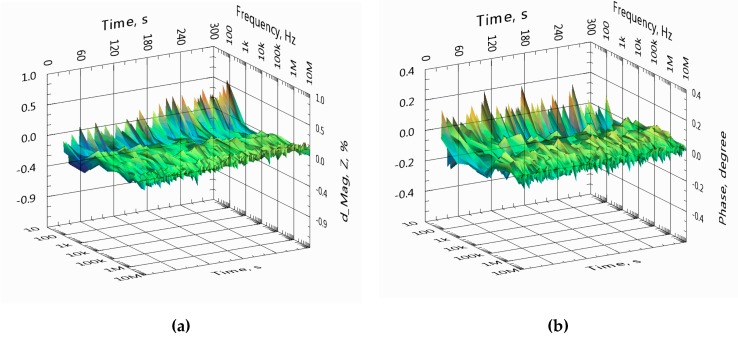
Overall variation of the impedance magnitude (**a**) and phase (**b**) of spectra during 5 min. The spectral data are collected from the same sensor and at the same timeframe as presented in Figure 15 for the single frequency of 10 kHz.

**Figure 15:**
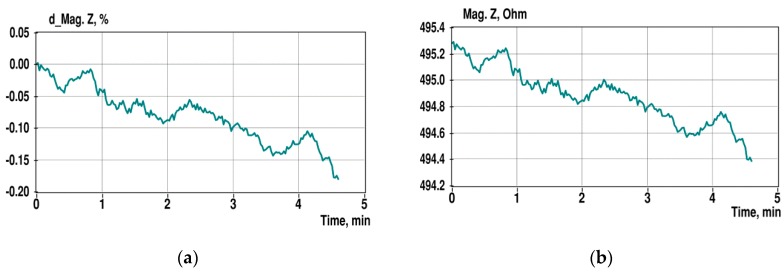
Relative (**a**) and absolute (**b**) value changes of the impedance magnitude at 10 kHz during 5 min observation. Additional moving average filtration is used to suppress high-speed fluctuations.

In Figure 14, the overall variations of the impedance spectra are shown in the same timeframe as presented in Figure 15 but without additional filtering. Note that the variation of the magnitude spectrum increases at the lower frequency end (100 Hz) and grows somewhat higher at 10 kHz, since additional filtering is not applied here. Relative standard deviation (RSD) of impedance magnitude at 10 kHz is 0.07% (without additional filtering).

In comparison with the results of the previous experiment, see Figure 10, the relative change of the impedance magnitude is around seven times less, and it decreases slightly at higher frequencies. However, RSD of the impedance magnitude at 10 kHz remains close at 0.07% against 0.09% for the previous experiment.

### 4.4. Measurement Results with a Sensor using “Platypus” Electrodes

In this experiment, the sensors with ultra-flat gold-plated glass chips AU.1000.STWG from Platypus Technologies LLC (Fitchburg, WI, USA) were used. According to the provided data, the RMS of the roughness of the gold-plated surface of chips is only 3.6 Angstroms (0.36 nm).

**Figure 16:**
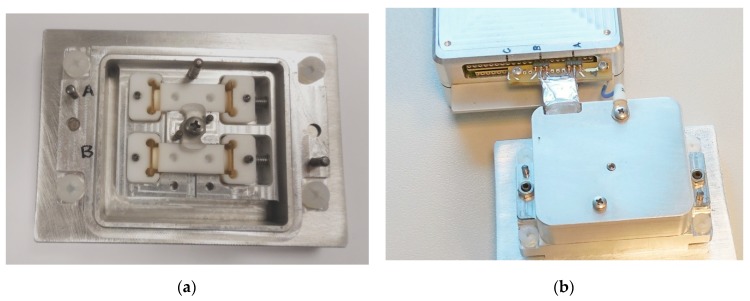
Two sensors in the aluminum base part (**a**) and sensors unit connected to the EIS analyzer (**b**).

Two sensors with cylindrical measurement chambers are placed in the aluminum compartment, as shown in Figure 16. Electrode surfaces are connected to the connector leads with the aid of spring contacts adjusted with a screw in the center of the top part. The diameter of the sensor chambers is 4 mm, and the distance between electrode surfaces is 24 mm.

**Figure 17:**
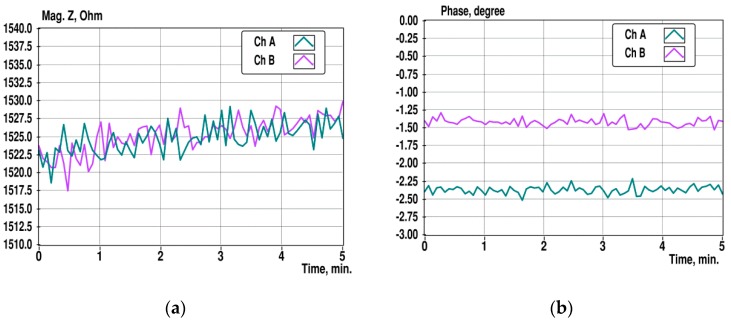
Changes of the impedance magnitude (**a**) and phase (**b**) at 10 kHz during 5 min observation in sensor channels A and B filled with PBS.

In Figure 17, the change of the impedance spectra in time at a single frequency of 10 kHz is shown for a PBS solution in sensor channels A and B. The excitation has nine frequencies ranging from 100 Hz to 10 MHz, and the data of 72 impedance measurements were collected within 4.2-s intervals over 5 min. LINDY’s (LINDY International Limited, Thornaby, UK.) fibre optic extender (see Section 2.5.) was used for galvanic isolation of the USB port of the analyzer in this experiment.

There is no significant transition in the impedance settling process observed for these measurements. The slow increase of the magnitudes in both channels (around 0.3 %) may be caused by evaporation or change of temperature. Note that the common aluminum enclosure (Figure 16) ensures lesser differences in sensor temperatures. The difference of phases is caused by inaccuracy of calibration, which is discussed in the next section.

**Figure 18:**
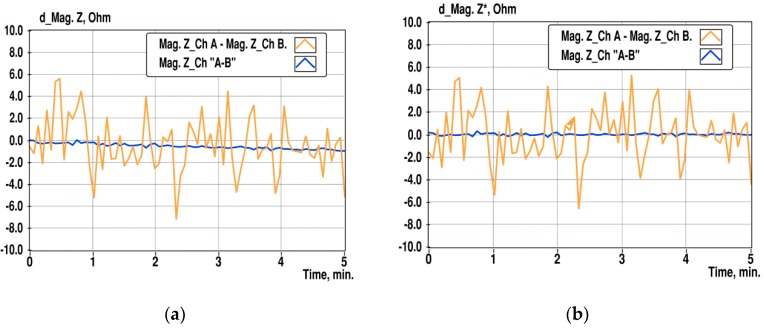
Change of the difference of the impedance magnitudes in channels A and B at 10 kHz, and in the differential channel “A-B” during 5 min of observation (**a**), and linearized differences of the same data (**b**).

The difference of impedances obtained by subtraction of results from channels A and B, d_Mag Z, and from differential channel “A-B,” d_Mag Z*, as a magnitude of the complex impedance calculated according to Equation (4) are as small as illustrated in Figure 18a. After straightening curves using a linear fit with the least-squares method, the calculated STD of d_Mag Z is 2.4 Ω, and 0.1 Ω for d_Mag Z*. Corresponding variances are 6.0 and 0.01, respectively.

Relative standard deviation (RSD) of d_Mag Z against the mean impedance magnitude in channel A is 0.03%, and 0.006% for d_Mag Z*, obtained from differential channel measurement.

In comparison with the results of previous experiments (see Figure 15), the relative change of impedance magnitude is similar. However, RSD of the impedance magnitude at 10 KHz is more than two times less than for direct channel measurements. At the same conditions, the RSD of impedance magnitude changes d_Mag Z* obtained from the differential channel is near to five times less in comparison with the first experiment (see Section 4.1).

**Figure 19:**
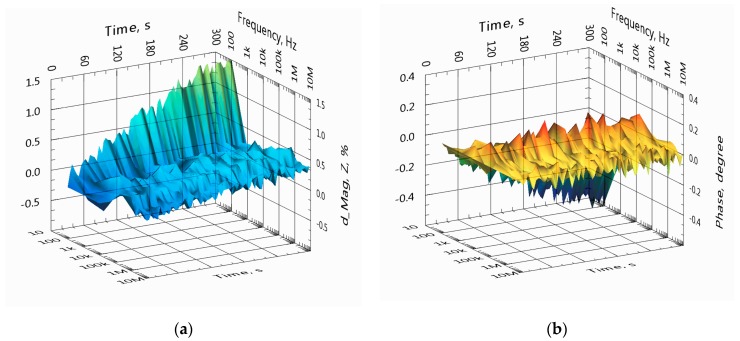
Overall variation of the impedance magnitude (**a**) and phase (**b**) of spectra over 5 min. The spectra are collected from the same sensor and at the same timeframe as presented in Figure 17 for the single frequency of 10 kHz.

In Figure 19, the overall variation of the impedance spectra is shown in the same timeframe as presented in Figure 17.

The main measurement results focusing on the detection of impedance changes with different sensors are presented in Table 1. Mean values of the impedance magnitude |Z|_mean are calculated from the measurement results of channel A at 10 kHz during an observation time of 5 min. Here, notation d_|Z| stands for the difference of impedance magnitudes found by subtracting the measurement results from channels A and B, and d_|Z|* stands for the magnitude difference obtained from differential channel “A-B” using a magnitude of the complex impedance calculated according to (4).

It may be concluded that the use of the differential channel allows a significant decrease in the relative standard deviation of the measurement results, but the smoothness of electrodes is also a very important factor. There are several methods and technologies for producing smooth electrode surfaces, e.g., template-stripping [23], chemical vapor transport (CVT) [24], pressure-induced surface deformation [25], and chemical mechanical polishing (CMP) [26].

### 4.5. Calibration Issues.

As shown in Figure 8, both the magnitude and phase have a rising shape at frequencies above 100 kHz before initial calibration. The main reason for such effect is a degradation of the performance (lower gain and higher phase lag) of the response signal chain (mainly in TIA) at higher frequencies and due to the influence of stray capacitances. As discussed in Section 4.1, calibration with a precision resistor does not compensate those changes fully when measuring the impedance of more complex objects. One reason is that the transfer characteristic of the op-amp depends on the signal amplitude and this dependence increases along with frequency. The degradation can be reduced by decreasing the gain of TIA. However, this also degrades the overall sensitivity. The second possibility is to use a more sophisticated calibration procedure with the aid of reference elements, which are more similar to the SUT to be measured.

**Figure 20:**
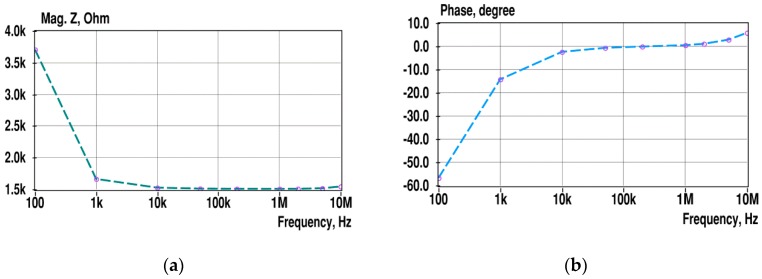
Mean magnitude (**a**) and phase (**b**) spectra of the PBS solution measured with “Platypus” sensors described in Section 4.3. Ring-shaped marks indicate the used frequencies.

Another calibration issue is illustrated in Figure 20.

**Figure 21:**
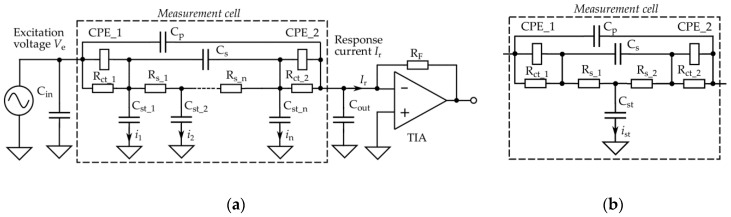
Measurement circuitry with more detailed electrical model of the measurement cell (**a**) and its simplified variant (**b**).

Obtained spectra do not fit with the expected decaying shape of spectra at higher frequencies, as described in the introduction part (see Figure 2), and the cause of such distortions is an important question. The answer is that in the higher frequency area, the impedance model of precision resistors used for initial calibration does not match fully with the parameters of used practical sensors and measurement circuitry. In practical sensors, not all of the response current reaches the input of its measurement circuitry; in our case, inputs of the transimpedance amplifiers U1 to U3 (see Figure 3). The reason is that a part of the current, the sum of *i*_1_ to *i*_n_, spreads through the stray capacitances C_st(*i*)_, as illustrated in Figure 21.

In the strict electrical model, the resistance of the solution R_s_ and stray capacitances C_st_ both must be treated as an electrical circuit with distributed parameters. The input and output capacitances C_in_, C_out_, and C_p_, connected in parallel with the measurement cell, also influence the response current *I*_r_. C_s_ is a capacitance of the solution between electrodes, and R_ct_ denotes so-called charge-transfer resistance that is in parallel with the constant-phase elements (CPE). However, in most practical cases, the electrical model of the measurement cell may be simplified, as illustrated in Figure 21b. Our preliminary tests show that the division of the R_s_ C_st_ pairs into two sub-circuits did not improve fitting accuracy of other model element values (in the relative error range of ± 0.1%).

The influence of the input capacitance C_in_ is negligible because of the low output impedance of the excitation source. The influence of C_out_ and C_p_ is also small since the input voltage of TIA is kept near zero by the negative feedback. Moreover, in our case, the variations of the excitation signal are compensated by its synchronous measurement.

Nevertheless, a more accurate detection of electrical parameters of SUT requires additional measurements in the higher frequency area. The influence of C_st_ must be known beforehand, as otherwise, it is impossible to exactly compensate the distortions it produces. One solution is to determine the values of C_st_, R_s_1_, and R_s_2_ with a test liquid (e.g., PBS solution) that is as similar as possible to that used in the subsequent experiments. Obtained values will be later used for the correction of measured response current spectra. Another possibility is a modification of the calibration reference ${\vec{Z}}_{r}$ parameters in Equation (3) so that they produce the expected spectra of the test liquid. The accuracy of both corrections depends on the properties of the measurement cell and test liquid. The analysis of the error level dependence on the variation of these properties is a topic of further research.

### 4.6. Topics of Further Research and Enhancement.

The solution to calibration problems requires further studies. The best solution for reducing the influence of discussed stray capacitances is their minimization. A short connection to the front-end electronics in a medium with low permittivity provides said minimization.

Another topic requiring further research is the reliable detection of the polarity (direction) of small impedance changes at the output of the differential channel. Currently, this data is obtained by a comparison of impedance magnitudes of channels A and B. However, the results of this comparison may be distorted in the cases where the properties of SUT change during the measurement cycle.

The battery charge level should be monitored and a low battery message sent out via GUI.

## 5. Conclusions

The introduced compact and inexpensive triple channel EIS analyzer uses a near-to simultaneous direct and differential method of measurement that provides sensitive detection of impedance changes without losing information about their actual values.

The proposed novel design of the sensor with spatial electrodes provides tiny measurement cells without significant increase of the impedance of electrodes, which allows increased sensitivity of detection of small particles and their properties.

Experiments with the introduced novel and other sensors show that good smoothness of electrode surfaces is required to avoid long and volatile settling processes of impedance spectra. It was also observed that smooth electrodes provide a significantly lesser relative standard deviation (RSD) of impedance measurement results.

Calibration issues, including the influence of stray capacitances, are discussed in the last section, and several topics that require further research are pointed out.

## 6. Patents

Tallinn University of Technology has a pending patent application (P201900007) that details the novelties of the design of the microfluidic sensor introduced in this paper.

## Figures and Tables

**Table 1:** Summary of impedance magnitude measurement results with different sensors.

Sensor Type (Section No.)	|Z|_mean [Ohm]	STD d_|Z| [Ohm]	STD d_|Z|* [Ohm]	RSD d_|Z| %	RSD d_|Z|* %
4.1	28360	23.5	7.32	0.085	0.026
4.2	494.9	0.33 ^1^	n/a	0.067 ^1^	n/a
4.3	1524.9	2.44	0.096	0.029	0.006

^1^ STD and RSD are obtained here using values from channel A only.

## Appendix B.

The magnitude and phase spectra of the dummy cell, containing parallel connection of a 2 kΩ resistor and a 10 pF capacitor in series with a 10 nF capacitor (SUT1), was measured with the developed analyzer (AN) and the commercial precision impedance analyzer Wayne-Kerr 6500B (WK). The obtained measurement results were then compared with the impedance spectra of the ideal model of SUT1 with nominal values of its electrical components.

The relative error of the impedance magnitude and difference in phases is obtained by comparison of the mean values of collected results over 5 min. The excitation voltage amplitude was ± 50 mV for both instruments.

In Figure A8, Figure A9, Figure A10 and Figure A11 the magnitude and phase spectra of dummy-cell-containing parallel connection of a 10 kΩ resistor and a 10 pF capacitor in series with a 10 nF capacitor (SUT2) are shown, which were measured similarly to those in a previous case with SUT1. The same initial calibration was also used.
